# Preliminary evidence for a lower brain age in children with attention-deficit/hyperactivity disorder

**DOI:** 10.3389/fpsyt.2022.1019546

**Published:** 2022-12-02

**Authors:** Florian Kurth, Jennifer G. Levitt, Christian Gaser, Jeffry Alger, Sandra K. Loo, Katherine L. Narr, Joseph O’Neill, Eileen Luders

**Affiliations:** ^1^School of Psychology, University of Auckland, Auckland, New Zealand; ^2^Division of Child and Adolescent Psychiatry, Semel Institute for Neuroscience and Human Behavior, University of California, Los Angeles, Los Angeles, CA, United States; ^3^Department of Psychiatry and Psychotherapy, Jena University Hospital, Jena, Germany; ^4^Department of Neurology, Jena University Hospital, Jena, Germany; ^5^Department of Neurology, University of California, Los Angeles, Los Angeles, CA, United States; ^6^Brain Research Institute, University of California, Los Angeles, Los Angeles, CA, United States; ^7^Laboratory of Neuro Imaging, School of Medicine, University of Southern California, Los Angeles, CA, United States; ^8^Department of Women’s and Children’s Health, Uppsala University, Uppsala, Sweden

**Keywords:** ADHD, brain, BrainAGE, development, machine learning, relevance vector, sex, siblings

## Abstract

Attention-deficit hyperactivity disorder (ADHD) is a debilitating disorder with apparent roots in abnormal brain development. Here, we quantified the level of individual brain maturation in children with ADHD using structural neuroimaging and a recently developed machine learning algorithm. More specifically, we compared the BrainAGE index between three groups matched for chronological age (mean ± SD: 11.86 ± 3.25 years): 89 children diagnosed with ADHD, 34 asymptomatic siblings of those children with ADHD, and 21 unrelated healthy control children. Brains of children with ADHD were estimated significantly younger (−0.85 years) than brains of healthy controls (Cohen’s *d* = −0.33; *p* = 0.028, one-tailed), while there were no significant differences between unaffected siblings and healthy controls. In addition, more severe ADHD symptoms were significantly associated with younger appearing brains. Altogether, these results are in line with the proposed delay of individual brain maturation in children with ADHD. However, given the relatively small sample size (*N* = 144), the findings should be considered preliminary and need to be confirmed in future studies.

## Introduction

Attention-Deficit/Hyperactivity Disorder (ADHD) is a neurodevelopmental disorder that affects approximately 5% of all children and adolescents worldwide, with a higher incidence in males compared to females (1–5). ADHD has been widely reported to be associated with smaller global, regional, and local brain measures, both on the cortical and subcortical level and with respect to both gray matter and white matter (1–3, 6–25). Importantly, such effects were not only reported in classic analyses of data obtained on a single site, but also in meta- and mega-analytic analyses in very large samples from multiple sites (13–19). Moreover, there is evidence from longitudinal studies for lagging trajectories of brain development in ADHD (20–22). Altogether, these findings have been interpreted as a neurodevelopmental delay in ADHD (2, 3, 11, 26, 27), which in turn might be reflected in a seemingly younger brain age.

To our knowledge, only two other studies (28, 29) have investigated brain age in ADHD and, indeed, one observed younger appearing brains in children and adolescents with ADHD albeit not significantly so (28). The other one observed older appearing brains in participants with ADHD but findings were not significant either (29). However, no brain age study was conducted with explicit focus on ADHD (the main motivation of the former study was to evaluate a new brain age classifier; the latter study investigated multiple psychiatric disorders), so further research in the framework of ADHD is clearly indicated. On this note, ADHD has a strong genetic component, with a heritability of over 70% and a 5–10-fold increased risk for siblings to develop ADHD (1–3, 30–33). Given that previous studies reported that unaffected siblings of children with ADHD had similar but less pronounced gray matter alterations than their affected siblings (24, 25), it would also be interesting to explore if a delayed brain maturation is similarly evident in the healthy siblings of the children with ADHD.

Therefore, to shed further light on these questions and contribute to an understudied field of research, here we applied a well-validated brain age estimation algorithm (34–39) in children with ADHD, their healthy siblings, as well as age-matched unrelated control children. We hypothesized that children with ADHD will be estimated younger (i.e., their BrainAGE index will be lower) than age-matched controls, in accordance with the outcomes of the majority of the aforementioned studies (1–3, 6–27) as well as with the classification of ADHD as a neurodevelopmental disorder (2, 3, 11, 26, 27). Moreover, we hypothesized negative correlations between individual ADHD symptoms and estimated brain age (i.e., the more severe the symptoms, the more negative the BrainAGE index). As far as the siblings (who share genetic material with the ADHD children) are concerned, we hypothesized them to have younger appearing brains than the age-matched controls as well, but that these differences will be smaller than when comparing the ADHD children to the controls (i.e., ADHD children < unaffected siblings < healthy controls).

## Materials and methods

### Participants

A total of 144 participants (ADHD children, unaffected siblings, and control children) were recruited from the community, drafted from other ongoing studies of ADHD at the University of California, Los Angeles (UCLA), or referred to the study by their physicians. All children were evaluated for ADHD and other psychiatric disorders based on an interview with their primary caretaker using the Kiddie-Schedule for Affective Disorders and Schizophrenia–Present and Lifetime (K-SADS-PL) (40). In addition, direct interviews were performed with children who were 8 years and older. To supplement the interview(s), parent ratings on the Swanson, Nolan, and Pelham, Version IV (SNAP-IV) Rating Scale (41) were obtained. Children with ADHD were enrolled together with their siblings. If the siblings met the full diagnostic criteria for ADHD, they were enrolled in the ADHD group; if they did not meet diagnostic criteria for ADHD, they were enrolled as unaffected siblings. Unrelated children who did not meet diagnostic criteria for ADHD or any other psychiatric disorder were enrolled as control children; all of those were singletons. Exclusion criteria for all children (ADHD, siblings, and controls) were neurological disorders, significant head injuries, premature birth (≤34 weeks gestation), low IQ (<70 Full-Scale), lifetime diagnosis of schizophrenia or of autism, and any contraindications to magnetic resonance imaging (MRI). Overall this resulted in the inclusion of 89 children with ADHD, 34 siblings without ADHD (hereafter referred to as siblings), and 21 controls. Table 1 provides an overview on the sample characteristics. Importantly, there were no significant differences in chronological age across the three groups (*p* = 0.363). However, the distribution of boys and girls differed significantly between the three groups (*p* < 0.001), with a significantly higher male-to-female ratio in children with ADHD compared to siblings and controls. All participants provided written informed consent/assent and the study was approved by the UCLA Institutional Review Board. Additional approval for the data analysis was obtained from the University of Auckland Health Research Ethics Committee.

**TABLE 1:** Sample characteristics.

	Controls	Siblings	ADHD
Sample size	21	34	89
Age [years; mean ± SD (range)]	12.7 ± 2.2 (8.6–15.3)	11.4 ± 3.5 (6.5–18.9)	11.9 ± 3.3 (6.1–18.8)
Sex*	7 boys/14 girls^a^	9 boys/25 girls^a^	58 boys/31 girls^c,s^
Hyperactivity score–Parent SNAP^#^ [mean ± SD (range)]	0.1 ± 0.2 (0.0–0.7)^a,s^	0.4 ± 0.4 (0.0–1.7)^a,c^	1.7 ± 0.7 (0.0–3.0)^c,s^
Inattention score–Parent SNAP^#^ [mean ± SD (range)]	0.3 ± 0.3 (0.0–0.8)^a,s^	0.5 ± 0.5 (0.0–2.2)^a,c^	2.1 ± 0.6 (0.4–3.0)^c,s^
Combined score–Parent SNAP^#^ [mean ± SD (range)]	0.2 ± 0.2 (0.0–0.6)^a,s^	0.5 ± 0.5 (0.0–1.9)^a,c^	1.9 ± 0.6 (0.4–3.0)^c,s^
Number of hyperactive symptoms^#^ [mean ± SD (range)]	0.1 ± 0.2 (0.0–1.0)^a^	0.4 ± 0.8 (0.0–4.0)^a^	4.8 ± 2.8 (0.0–9.0)^c,s^
Number of inattentive symptoms^#^ [mean ± SD (range)]	0.1 ± 0.2 (0.0–1.0)^a,s^	1.0 ± 1.3 (0.0–4.0)^a,c^	7.4 ± 1.7 (1.0–9.0)^c,s^

*The distribution of boys and girls differed significantly between the groups (*p* < 0.001) as determined using a chi^2^ test.

^#^The symptoms differed significantly between the groups (*p* < 0.001) as determined using permutation tests.

^a^This group differs significantly from the ADHD group (*p* < 0.05).

^c^This group differs significantly from the control group (*p* < 0.05).

^s^This group differs significantly from the sibling group (*p* < 0.05).

### Data acquisition and preprocessing

All brain images were acquired on a 3T Siemens Trio Scanner at the UCLA Ahmanson-Lovelace Brain Mapping Center using a 21-channel head coil and the following parameters: *TR* = 1,900 ms, *TE* = 3.26 ms, flip angle = 9°, 176 axial sections, voxel size = 0.98 mm × 0.98 mm × 1 mm. These T1-weighted images were then processed in Matlab^1^ using SPM12^2^ and the CAT12 toolbox (42). As detailed elsewhere (35, 36, 38, 39), all images were corrected for magnetic field inhomogeneities, tissue-classified into gray matter, white matter, and cerebrospinal fluid, spatially normalized to MNI space using affine transformations and convoluted using a Gaussian Kernel. These processed images provided the input for the BrainAGE analysis (see next section).

### Estimating brain age

The BrainAGE approach used in our study was specifically validated in the context of neurodevelopment (35) and shown to be robust and reliable across datasets, age ranges, and scanner types (34, 36). The algorithm is based on a Relevance Vector Regression Machine (35, 43), and has been successfully applied in a number of studies (e.g., 34, 37, 38, and 39). The current study used the same workflow as described elsewhere (34, 35), but the model was trained on an even larger sample of 879 healthy children and adolescents aged 5–22 years (mean age 12.3 years, NIH Pediatric MRI Data Repository 4th release). This trained algorithm was applied to the processed gray matter images of the current sample (see previous section) to estimate each brain’s age. The difference between the estimated age and the chronological age yielded the brain age gap estimate (BrainAGE) index (see Supplementary Figure 1 for further details). The BrainAGE index is negative if a brain is estimated younger than its chronological age (positive if older than its chronological age). Thus, a negative BrainAGE index is consistent with a delay in brain maturation. Importantly, to ensure that the BrainAGE index is not correlated with age, any links between the two variables were removed (44, 45) as described elsewhere (37).

### Statistical analyses

#### Main analyses

To compare BrainAGE across the three groups, the BrainAGE indices were the dependent variable and group status (i.e., ADHD, siblings, and controls) was the independent variable. Sex was included as a covariate because the distribution of boys and girls differed between the groups, and “family” was included as a random variable because several of the participants were siblings. In addition, we tested whether there was a significant group-by-sex interaction because the prevalence of ADHD during childhood and adolescence is higher in males than in females (4). However, the inclusion of a group-by-sex interaction did not improve the model (*p* = 0.332) but, instead, increased the Akaike Information Criterion suggesting that the model without the interaction term was a better fit. Moreover, the interaction did not reach significance [*F* (2,138) = 1.11, *p* = 0.332]. Thus, the model was conducted without the group-by-sex interaction. Given our hypothesis that children with ADHD as well as their siblings (albeit to a lesser degree) would present with a delay in brain maturation (i.e., younger appearing brains) compared to control children, one-tailed *T*-tests were applied. Assumptions for parametric testing were confirmed using a Lilliefors test determining the normal distribution of the residuals and a Bartlett test determining equality of variance.

To investigate correlations between BrainAGE and measures of symptom severity, the BrainAGE indices were the dependent variable, the five symptom measures were the independent variable (a separate model was run for each), sex was a covariate, and “family” was a random variable. Again, the addition of a sex interaction did not improve the models and also did not reach significance for any of the symptom measures (see Supplementary Table 1); it was thus omitted. The aforementioned five symptom measures were (1) the Parent SNAP-IV hyperactivity score, (2) the Parent SNAP-IV inattention score, (3) the Parent SNAP-IV combined score, (4) the number of hyperactivity symptoms, and (5) the number of inattention symptoms. Eight participants (one control, four siblings, and three ADHD children) did not have scores for the SNAP, and three controls did not have scores for the number of symptoms and were therefore excluded from the correlation analyses. Given our hypothesis of a negative correlation between the number of symptoms and brain maturation (the more symptoms, the younger the brain), one-tailed *T*-tests were applied. Given the five different models for the five different symptom scores, corrections for multiple comparisons were applied using the false discovery rate (FDR) (46). Assumptions for parametric testing were confirmed using Lilliefors tests determining the normal distribution of the residuals.

#### Supplemental analyses

No significant sex interaction was found, neither in the group comparisons nor the correlation analyses. However, given the higher prevalence of ADHD in males during childhood and adolescence (4), we conducted an additional set of exploratory analyses repeating the main analyses described above in males and females separately. Moreover, we repeated the correlation analyses for both sexes combined but only in children with ADHD.

## Results

### Main findings

**FIGURE 1:**
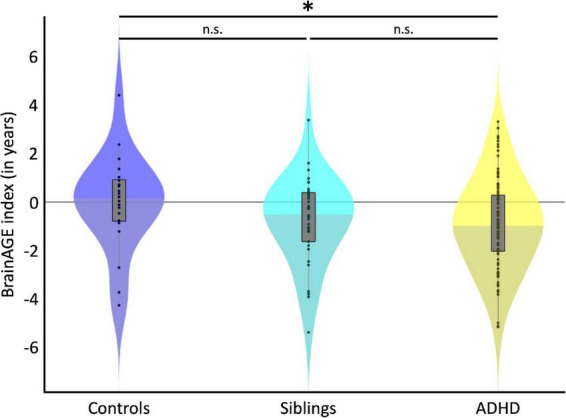
Group Differences. Violin plots depict the BrainAGE distribution for each group. The black dots show individual estimates, the gray boxes show the group-specific interquartile ranges, and the whiskers show the group-specific 1.5 interquartile ranges. Shading for each group’s violin plot changes at the median. On average, children with ADHD (yellow) were estimated significantly younger than control children (blue), as indicated by the asterisk. Siblings unaffected by ADHD (cyan) were also estimated younger than control children (blue), but this difference did not reach statistical significance (n.s.).

Figure 1 depicts the individual and group-specific BrainAGE indices with negative numbers indicating a delay in brain maturation (i.e., brains are estimated younger than their chronological age). On average, the BrainAGE index was 0.0 ± 1.95 years in controls (blue), −0.71 ± 1.78 in siblings (cyan), and −0.85 ± 1.87 in children with ADHD (yellow). There was a delay in brain maturation of 0.71 years (which corresponds to 8.5 months) in siblings and of 0.85 years (which corresponds to 10.2 months) in children with ADHD. The delay in brain maturation did not reach significance in siblings compared to controls [Cohen’s *d* = −0.23, *t* (140) = −1.38, *p* = 0.086], but was significant for children with ADHD compared to controls [Cohen’s *d* = −0.33, *t* (140) = −1.93, *p* = 0.028]. The difference in brain maturation between children with ADHD and siblings was not significant [Cohen’s *d* = −0.08, *t* (140) = −0.49, *p* = 0.313].

**FIGURE 2:**
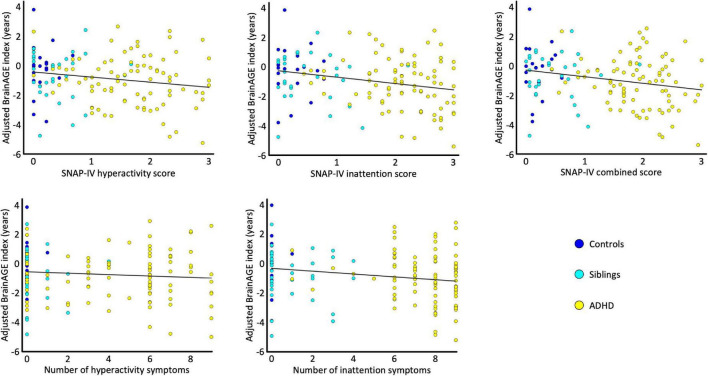
Correlations. The scatterplots depict the association between the sex-adjusted BrainAGE index and the SNAP-IV hyperactivity scores, the SNAP-IV inattention scores, the SNAP-IV combined scores, the number of hyperactivity symptoms, and the number of inattention symptoms. For each scatterplot the estimated association is plotted as a least-square regression line. All data points are color-coded by group (control children in blue, unaffected siblings in cyan, and children with ADHD in yellow).

Figure 2 depicts the link between the individual BrainAGE indices and symptom scores. All correlations were negative indicating that a lower BrainAGE index is associated with more severe symptoms. As detailed in Table 2, the correlations were significant for all symptom severity scores, except for the number of hyperactivity symptoms.

**TABLE 2:** Links between BrainAGE index and ADHD symptom severity.

Behavioral measures	Correlation coefficient	*T* (df) statistics	*p*-statistics
Hyperactivity score–Parent SNAP	*r* = −0.16	*T* (133) = −1.88	*p* = 0.031*
Inattention score–Parent SNAP	*r* = −0.21	*T* (133) = −2.51	*p* = 0.007*
Combined score–Parent SNAP	*r* = −0.20	*T* (133) = −2.36	*p* = 0.010*
Number of hyperactivity symptoms	*r* = −0.08	*T* (138) = −0.94	*p* = 0.174
Number of inattention symptoms	*r* = −0.18	*T* (138) = −2.18	*p* = 0.016*

*Survives FDR correction.

### Supplemental findings

The exploratory analyses investigating group differences and associations with symptom scores in boys and girls separately suggest that effects are primarily found in boys. However, given that group-by-sex interactions and symptom-by-sex interactions in the main analyses were not significant, these findings will solely be provided as a reference for future studies without interpreting them (see Supplementary Tables 2, 3). When confining the correlation analyses to the ADHD group, the associations remained negative for all but the number of hyperactivity symptoms but only reached significance (albeit uncorrected) when stratified by sex. Similarly to the sex-stratified group differences and correlations in the whole group, these findings are also provided solely as a reference (see Supplementary Table 4).

## Discussion

Originally catalyzed by a competition (47), a number of studies have used machine learning tools to predict the diagnosis of ADHD based on MRI scans of the brain (48). Deriving at a diagnosis for ADHD based on machine learning (or neuroimaging in general) still poses an ongoing challenge but has since led to promising advances in the field (48). Importantly, while the BrainAGE approach is also based on machine learning it was not designed to diagnose ADHD. Instead, it was applied to test for a potentially delayed brain maturation in ADHD. More specifically, this was achieved by estimating the individual brain ages and calculating their deviation from the chronological ages—revealing the BrainAGE indices (34–39)—which were then compared between children with ADHD and age-matched controls as well as related to ADHD symptoms. We detected significantly younger appearing brains in children with ADHD compared to age-matched controls and we also observed significant negative links between ADHD symptom severity and BrainAGE. Altogether, these findings are in strong agreement with the majority of previous observations (1–3, 6–28), suggesting a neurodevelopmental delay in ADHD (2, 3, 10, 11, 26, 27). Moreover, as deviations from normal brain development have been proposed as defining traits of neurodevelopmental disorders (26, 27), the present results also support the classification of ADHD as a neurodevelopmental disorder (2, 3, 11, 26, 27). Of note, the direction of the currently observed effect is also in agreement with the outcomes of another brain age estimation study reporting younger brain ages in children with ADHD compared to controls (28), albeit the effect in that study was not significant. In contrast, the findings of the current study as well as most other existing findings (1–3, 6–25) seem to disagree with the outcomes of yet another brain age study, which revealed older brain ages in participants with ADHD (29), albeit effects were not significant either. Given the conflicting findings as well as the limited number of brain age studies overall, further research is clearly necessary.

In addition to children with ADHD and control children, the present study also included unaffected siblings of children with ADHD. The difference between siblings and controls did not reach significance, but the brains of siblings were estimated 8.5 months younger than the brains of controls. Given that the brains of children with ADHD were estimated 10.2 months younger than the brains of controls, the unaffected siblings were overall closer to their siblings with ADHD than to the controls. This finding seems to be consistent with the observation that ADHD runs in families, with siblings of children with ADHD having a 5–10-fold increased risk of developing ADHD when compared to the general population (2, 30, 31). It also corroborates the outcomes of previous imaging studies that revealed similar but less pronounces brain alterations in siblings (24, 25).

### Study limitations and implications for future research

Since the brain age estimation is a relatively novel approach, our study complements and extends the existing field of research. However, future studies are needed to replicate the present findings and address some study limitations: First of all, the sample size is rather small and would benefit substantially from an increase, particularly in the control group. This might not only enhance statistical power but also allow to stratify analyses by sex and to interpret sex-specific findings with confidence. For example, it is certainly noteworthy that our supplemental analyses revealed a lower BrainAGE index (compared to controls) only in boys with ADHD as well as in unaffected brothers, while no effects were evident in girls with ADHD or unaffected sisters. However, due to the non-significant sex-by-group interaction in the main analysis, which is potentially due to the limited statistical power, any interpretations would be conjecture. Moreover, while the current cross-sectional study captured apparent delays in brain maturation, future longitudinal studies may not only shed light on the causality of the effects but also on the onset as well as the longevity/stability of the effects. For example, ADHD has been reported to manifest in early childhood, middle childhood, adolescence, or even adulthood, where adulthood ADHD may or may not be a continuation of childhood ADHD (49–51). In the same vein, symptoms may increase, decrease, or stagnate over time (3, 26) and all this might be captured by changes in the BrainAGE index (or changes in brain anatomy in general). On that last note, it will certainly also be worthwhile finding a sustainable way to conduct more comprehensive studies in the field of ADHD linking a number of relevant aspects (i.e., brain structure, brain function, cognition, behavior, genes, and environment) rather than being restricted to one or two single aspects.

## Data availability statement

The data analyzed in this study is subject to the following licenses/restrictions: We do not have permission to share the data. Requests to access these datasets should be directed to FK, f.kurth@auckland.ac.nz.

## Ethics statement

The studies involving human participants were reviewed and approved by the UCLA Institutional Review Board and University of Auckland Health Research Ethics Committee. Written informed consent to participate in this study was provided by the participants’ legal guardian/next of kin.

## Author contributions

EL, FK, and JL planned the present analysis of the dataset, which was conceived, acquired, and curated by JL, SL, JO’N, JA, and KN. CG processed the MRI data and calculated the BrainAGE indices. FK conducted the statistical analyses. FK and EL wrote the manuscript. JO’N contributed to the writing and editing of the manuscript. All authors contributed to the article and approved the final manuscript.

## Footnotes

1
http://www.mathworks.com/products/matlab

2
http://www.fil.ion.ucl.ac.uk/spm
