# Supplementary material for: Preliminary evidence for a lower brain age in children with attention-deficit/hyperactivity disorder
Source: Front Psychiatry. 2022 Dec 2;13:1019546. doi: 10.3389/fpsyt.2022.1019546 (PMC9755736; doi:10.3389/fpsyt.2022.1019546)
Supplement: Supplementary file 1 [file Data_Sheet_1.PDF]

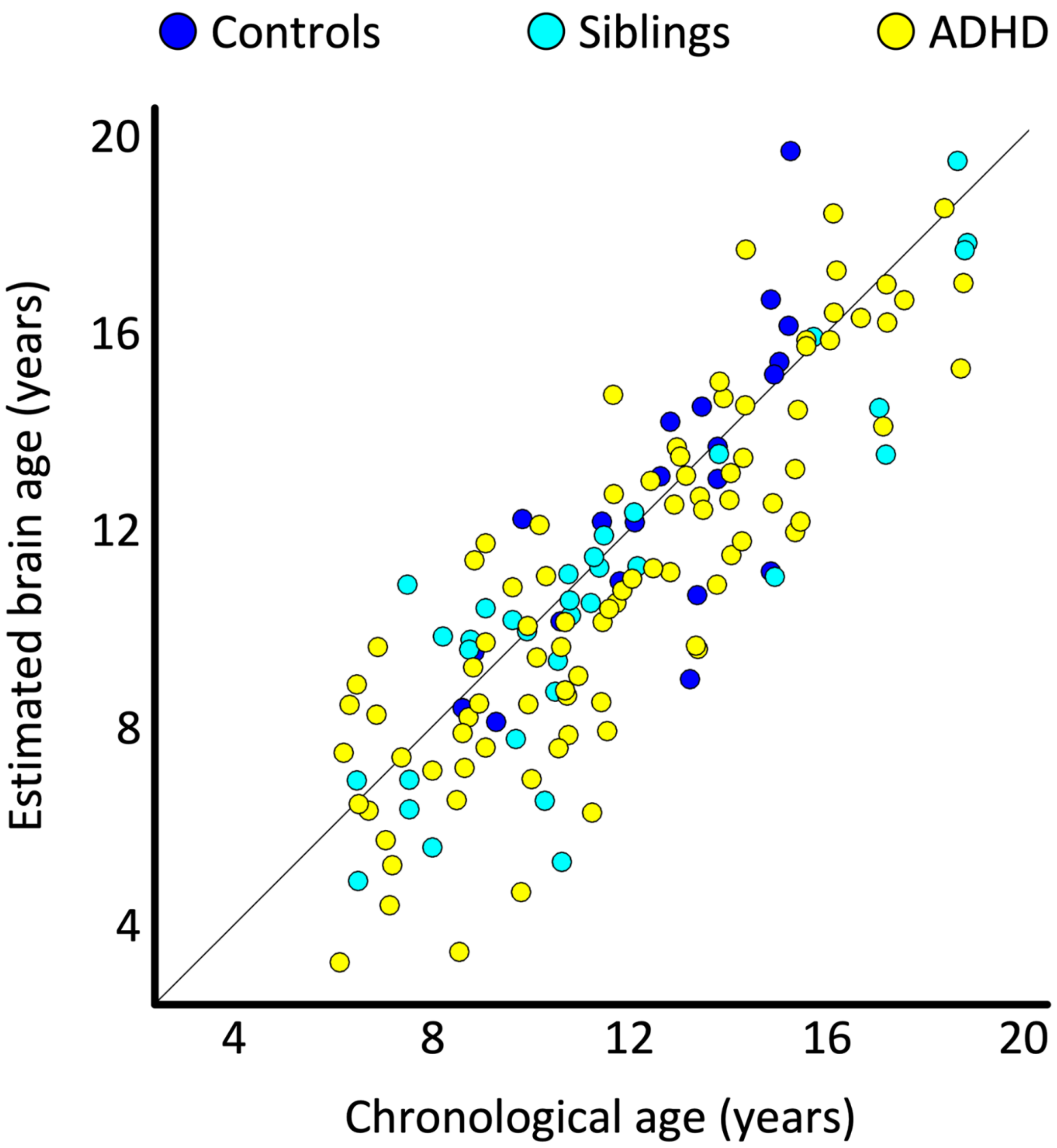

Supplementary Figure 1. Each participant's estimated age (y-axis) is plotted against their chronological age (x-axis). Each data point is color coded by group with controls in blue, unaffected siblings in cyan, and children with ADHD in yellow. The black line indicates the exact match of both estimated and chronological age. The mean absolute error of the estimated age is 1.36 years for controls, 1.37 years for unaffected siblings, and 1.64 years for children with ADHD. The BrainAGE index (i.e., the dependent variable in all analyses) is calculated as the difference between the estimated brain age and the chronological age.

Supplementary Table 1. Effects of adding a sex-by-symptom interaction term.

Behavioral Measures	Improvement of Statistical Model	Sex-by-Symptom Interaction	
Hyperactivity score - Parent SNAP	p=0.94	F(1,132)=0.01	p=0.938
Inattention score - Parent SNAP	p=0.12	F(1,132)=2.39	p=0.124
Combined score - Parent SNAP	p=0.35	F(1,132)=0.89	p=0.346
Number of hyperactivity symptoms	p=0.25	F(1,137)=1.34	p=0.249
Number of inattention symptoms	p=0.37	F(1,137)=0.81	p=0.371

The addition of a sex-by-symptom interaction term did not improve the statistical model. Moreover, the interaction term was not significant for any symptom scores.

Supplementary Table 2. Group differences for the BrainAGE index in boys and girls separately.

All Boys			
**Group Comparison**	**Cohen's d**	**t(df) statistics**	**p statistics**
Siblings vs. Controls	d=-0.43	t(71)=-1.81	p=0.037
ADHD vs. Controls	d=-0.49	t(71)=-2.04	p=0.022
ADHD vs. Siblings	d=0.06	t(71)=0.26	p=0.603
**All Girls**			
**Group Comparison**	**Cohen's d**	**t(df) statistics**	**p statistics**
Siblings vs. Controls	d=-0.08	t(67)=-0.33	p=0.372
ADHD vs. Controls	d=-0.22	t(67)=-0.90	p=0.186
ADHD vs. Siblings	d=-0.16	t(67)=-0.67	p=0.252

Supplementary Table 3. Links between the BrainAGE index and symptom severity in the complete sample, separately for boys and girls.

All Boys			
**Behavioral Measures**	**Correlation Coefficient**	**t(df) statistics**	**p statistics**
Hyperactivity score - Parent SNAP	r=-0.16	t(67)=-1.30	p=0.098
Inattention score - Parent SNAP	r=-0.31	t(67)=-2.65	p=0.018**
Combined score - Parent SNAP	r=-0.25	t(67)=-2.14	p=0.008**
Number of hyperactivity symptoms	r= 0.01	t(72)= 0.10	p=0.539
Number of inattention symptoms	r=-0.23	t(72)=-2.00	p=0.025**
**All Girls**			
**Behavioral Measures**	**Correlation Coefficient**	**t(df) statistics**	**p statistics**
Hyperactivity score - Parent SNAP	r=-0.17	t(65)=-1.39	p=0.084
Inattention score - Parent SNAP	r=-0.12	t(65)=-0.97	p=0.168
Combined score - Parent SNAP	r=-0.15	t(65)=-1.23	p=0.112
Number of hyperactivity symptoms	r=-0.22	t(65)=-1.85	p=0.034*
Number of inattention symptoms	r=-0.14	t(65)=-1.14	p=0.129

* uncorrected; **FDR-corrected

Supplementary Table 4. Links between the BrainAGE index and symptom severity in children with ADHD, combined and separately for boys and girls.

**All participants with ADHD**			
**Behavioral Measures**	**Correlation Coefficient**	**t(df) statistics**	**p statistics**
Hyperactivity score - Parent SNAP	r=-0.11	t(83)=-1.97	p=0.167
Inattention score - Parent SNAP	r=-0.16	t(83)=-1.51	p=0.067
Combined score - Parent SNAP	r=-0.16	t(83)=-1.46	p=0.074
Number of hyperactivity symptoms	r= 0.01	t(86)= 0.12	p=0.549
Number of inattention symptoms	r=-0.17	t(86)=-1.62	p=0.054
**Boys with ADHD**			
**Behavioral Measures**	**Correlation Coefficient**	**t(df) statistics**	**p statistics**
Hyperactivity score - Parent SNAP	r=-0.06	t(53)=-0.47	p=0.322
Inattention score - Parent SNAP	r=-0.27	t(53)=-2.03	p=0.024*
Combined score - Parent SNAP	r=-0.19	t(53)=-1.40	p=0.084
Number of hyperactivity symptoms	r= 0.11	t(56)=0.82	p=0.793
Number of inattention symptoms	r=-0.24	t(56)=-1.85	p=0.035*
**Girls with ADHD**			
**Behavioral Measures**	**Correlation Coefficient**	**t(df) statistics**	**p statistics**
Hyperactivity score - Parent SNAP	r=-0.30	t(29)=-1.70	p=0.050*
Inattention score - Parent SNAP	r=-0.14	t(29)=-0.74	p=0.232
Combined score - Parent SNAP	r=-0.24	t(29)=-1.36	p=0.093
Number of hyperactivity symptoms	r=-0.29	t(29)=-1.62	p=0.058
Number of inattention symptoms	r=-0.11	t(29)=-0.61	p=0.272

* uncorrected; **FDR-corrected
